# Mapping subcortical motor pathways in humans with startle-conditioned TMS

**DOI:** 10.1016/j.brs.2023.08.010

**Published:** 2023-08-17

**Authors:** Ronan A. Mooney, Amy J. Bastian, Pablo A. Celnik

**Affiliations:** a Department of Physical Medicine and Rehabilitation, Johns Hopkins University, School of Medicine, Baltimore, MD, USA; b Kennedy Krieger Institute, Baltimore, MD, USA; c Department of Neuroscience, Johns Hopkins University, School of Medicine, Baltimore, MD, USA

**Keywords:** Reticulospinal tract, Transcranial magnetic stimulation, Startle, Ipsilateral motor evoked potential

## Abstract

Subcortical motor pathways, such as the reticulospinal tract, are critical for producing and modulating voluntary movements and have been implicated in neurological conditions. Previous research has described the presence of ipsilateral motor evoked potentials (iMEPs) in the arm to transcranial magentic stimulation (TMS), and suggested they could be mediated by the uncrossed corticospinal tract or by ipsilateral cortico-reticulospinal connections. Here, we sought to elucidate the role of the reticulospinal tract in mediating iMEPs by assessing their modulation by a startling acoustic stimulus and mapping these responses across multiple upper limb effectors. In a first experiment, we delivered TMS at various intervals (1, 5, 10 and 15 ms) after a startling acoustic stimulus, known to excite the reticular formation, to elicit iMEPs in the arm. We observed robust facilitation of iMEP area when startle conditioning preceded TMS at the 10 ms interval. In a second experiment, we replicated our findings showing that both the area and number of iMEPs in the arm increases with startle conditioning. Using this technique, we observed that iMEPs are more prominent in the arm compared with the hand. In a third experiment, we also observed greater presence of iMEPs in flexor compared with extensor muscles. Together, these findings are consistent with properties of the reticulospinal tract observed in animals, suggesting that iMEPs primarily reflect reticulospinal activity. Our findings imply that we can use this approach to track modulation of cortico-reticulospinal excitability following interventions or neurological conditions where the reticulospinal tract may be involved in motor recovery.

## 1. Introduction

Subcortical motor projections are critical for producing and modulating voluntary movements, with the reticulospinal tract being one of the primary pathways [1]. Evidence from animal studies indicates that reticulospinal projections originate from the pontomedullary reticular formation in the brainstem [2], and span multiple segments in the spinal cord [3]. Studies in non-human primates have shown that the reticulospinal tract receives bilateral input from motor cortical areas [4,5], innervates motoneurons of multiple upper limb effectors bilaterally [6–8], primarily projects to proximal and flexor effectors [8–10], and is upregulated following lesions to the corticospinal tract [11,12]. Further, the reticulospinal tract is thought to underlie the startle response (i.e., rapid involuntary contraction of numerous muscles to a loud acoustic stimulus) [13,14], a reflex thought to have a protective function, preparing the body for a fight or flight reaction. Short latency responses (~3 ms) have been demonstrated in the pontomedullary reticular formation, but not other brainstem structures projecting to the spinal cord (e.g., vestibular nuclei), to startling acoustic stimuli [14]. However, despite an extensive understanding of the reticulospinal tract in animals, characterization of this pathway in humans has received less attention.

In humans, delivering transcranial magnetic stimulation (TMS) over primary motor cortex (M1) elicits contralateral and ipsilateral motor evoked potentials (cMEPs and iMEPs) in muscles of the contralateral and ipsilateral arms respectively [15]. While cMEPs primarily reflect the excitability of the crossed corticospinal pathway [16], the primary pathway mediating iMEPs is unclear. It has been proposed that iMEPs could be mediated by the small uncrossed portion (~10%) of the corticospinal tract [17], or by corticofugal fibers connecting with the ipsilateral reticular formation in the brainstem and then to motoneurons in the spinal cord (i.e., the reticulospinal tract) [15,18]. Elucidating the primary pathway mediating iMEPs has important implications for their utility as a non-invasive measure of reticulospinal function in humans.

Previous studies have demonstrated that delivering a startling acoustic stimulus 30–60 ms prior to TMS over M1 leads to suppression of cMEPs [19–22]. This suppresion is thought to reflect inhibition of motor cortical areas by ascending projections from the reticular formation via the thalamus [19]. In contrast, the effect of a startling stimulus on iMEPs has not been assessed. Raising the excitability of the reticular formation by delivering a loud acoustic stimulus at short latencies prior to TMS over ipsilateral M1 could shed light on the involvement of the reticulospinal tract in mediating iMEPs.

Here we sought to determine whether iMEPs are modulated by a startling acoustic stimulus and exhibit properties consistent with the reticulospinal tract in animals. We hypothesized that if iMEPs reflect reticulospinal activity, they, but not cMEPs, will be potentiated by startle conditioning. Based on non-human primate studies, we also hypothesized that if iMEPs are mediated by the reticulospinal tract, they would exhibit a proximal-to-distal gradient and show greater prominence in flexor muscles relative to extensors.

## 2. Materials and methods

### 2.1. Participants

Fourty-seven healthy young adults (26 females, mean ± SD age = 25 ± 5 years) participated in 3 separate experiments, with n = 10 in Experiment One, n = 22 in Experiment Two, and n = 15 in Experiment Three, and no participant overlap across them. We screened participants prior to enrollment for contraindications to TMS using a questionnaire. We assessed handedness (43 right, 4 left) using the Edinburgh Handedness Inventory [23]. The Johns Hopkins School of Medicine Institutional Review Board, in accordance to the declaration of Helsinki, approved the study and participants provided written informed consent.

### 2.2. Surface electromyography

We recorded surface electromyography (EMG) bilaterally from the *biceps brachii* (BB), *triceps brachii* (TB), *flexor carpi radialis* (FCR), *extensor carpi radialis* (ECR) and *first dorsal interosseous* (FDI) muscles using 25 mm square Ag–AgCl recording electrodes (Vermed, Buffalo, NY), arranged in a belly-tendon montage, with a ground electrode positioned on the lateral epicondyle of the humerus. EMG signals were amplified (1000 ×) and band-pass filtered (10–1000 Hz) using an AMT-8 amplifier (Bortec Biomedical Ltd, Alberta, Canada), sampled at 5 kHz using a Micro1401–4 interface (CED, Cambridge, England) and recorded with Signal software (Version 4.02; CED, Cambridge, England) for offline analysis.

### 2.3. Transcranial magnetic stimulation

**Fig. 1.**
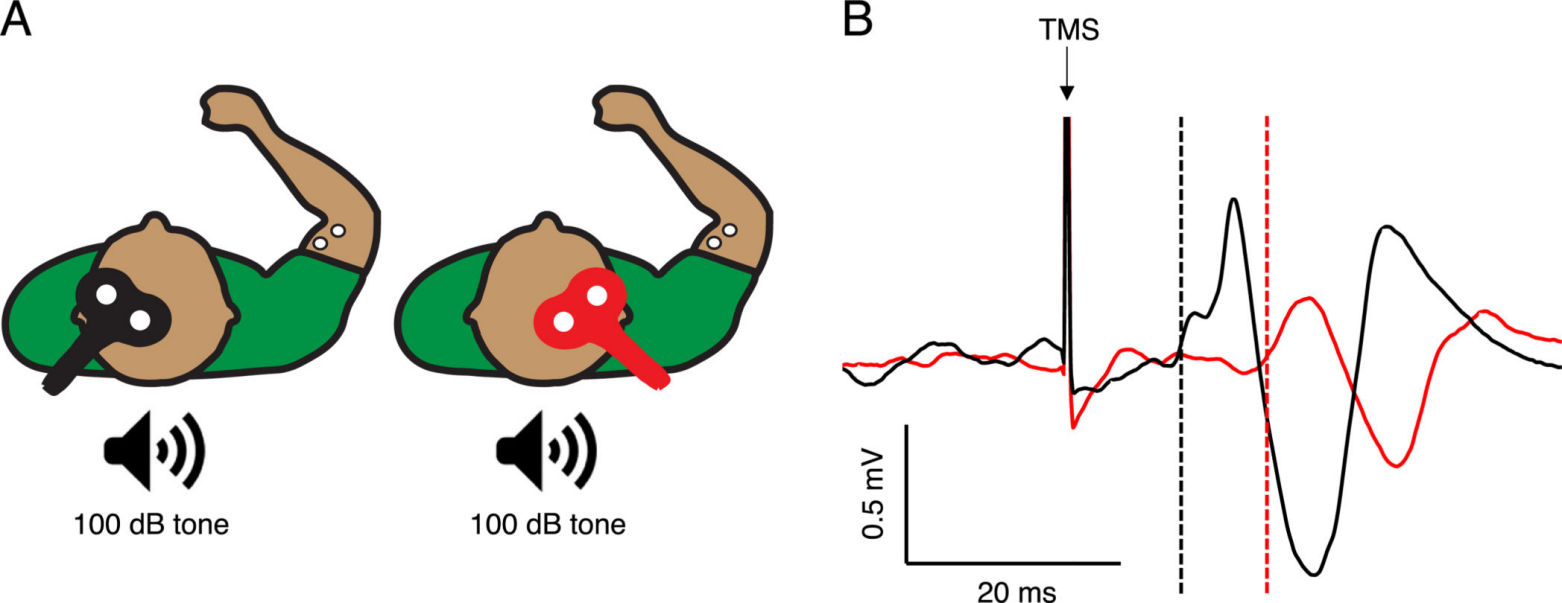
Experimental setup. (A) Transcranial magnetic stimulation (TMS) was delivered over the contralateral (black) and ipsilateral (red) primary motor cortex to the target biceps brachii muscle of the dominant arm. For the startle conditioning, a loud (100 dB) acoustic stimulus was presented through two speakers positioned behind the participant. (B) Example electromyography traces from an individual participant showing contralateral (black) and ipsilateral (red) motor evoked potentials to TMS alone in the biceps brachii muscle of the dominant arm. Dashed lines indicate onset latencies. (For interpretation of the references to colour in this figure legend, the reader is referred to the Web version of this article.)

Participants sat on a chair with their trunk straight, shoulders neutral, elbows at 90°, and forearms neutral. We applied single-pulse TMS to M1 bilaterally (Fig. 1A). A 70 mm-diameter figure-of-eight coil, connected to a monophasic Magstim 200^2^ magnetic stimulator (Magstim, Whitland, Wales), was held tangentially to the scalp (~45° to the mid-sagittal line) to induce posterior-anterior current in the brain. We identified the hot spot to elicit motor evoked potentials in the BB, TB, FCR, ECR and FDI of the dominant and non-dominant arm and hand by gradually moving the coil across the scalp to find the location which produced consistent responses in each muscle. The site of stimulation for each muscle and hemisphere was marked and kept constant throughout using Brainsight neuronavigation (Rogue Research, Montreal, Canada).

### 2.4. Experimental procedures

#### 2.4.1. Experiment One

In a first experiment, we tested whether combining a startling acoustic stimulus with TMS increases the size of iMEPs. We measured iMEPs) Fig. 1B in the dominant BB by delivering TMS over the ipsilateral M1 (non-dominant BB hotspot) at 100% maximum stimulator output with the target muscle pre-activated to ~20% of the participant’s maximum voluntary contraction (MVC) [15]. We delivered TMS alone or at 1, 5, 10 or 15 ms after the onset of a loud acoustic stimulus (frequency = 200 Hz, rise time = 1.6 ms, loudness = 100 dB). We chose these timing intervals based on the latency of the auditory brainstem reponse in humans (5–10 ms) [24], and the response latency in the pontomedullary reticular formation to startling acoustic stimuli in animals (~3 ms) [25]. We presented the acoustic stimulus through two speakers (Polk Audio, MD, USA) positioned ~30 cm behind the participant [21]. Each participant verbally confirmed that the acoustic stimuli were not painful and were tolerable. We visually observed a startle response in most participants upon initial presentation of the acoustic stimulus, which tended to habituate within a few trials as is commonly reported [13]. We delivered 12 trials for each condition (60 trials total) in a randomized order using Signal software (Version 4.02; CED, Cambridge, England). Next, we measured cMEPs in the dominant BB by delivering TMS over the contralateral M1 (dominant BB hotspot) at the intensity required to elicit an ~1.5 mV motor evoked potential with the target muscle pre-activated to ~20% of the participant’s MVC [26]. Again, we delivered 12 trials for each condition (60 trials total) in a randomized order. To control for a ceiling effect on the size of cMEPs, in a subgroup of participants (n =14; n =6 from Experiment One and n =8 from Experiment Two), we also measured the effects of startle conditioning (10 ms interval) on cMEPs at the TMS intensity required to elicit an ~0.5 mV motor evoked potential with the target muscle pre-activated to ~10% of the participant’s MVC. We delivered 12 trials for each condition (24 trials total) in a randomized order. For both cMEP and iMEP assessments, we provided visual feedback to the participant to assist maintenance of the target contraction. The inter-trial interval was 10 s to reduce anticipation of stimuli and 30 s rest breaks were provided after every four trials.

#### 2.4.2. Experiment Two

In a second experiment, we sought to replicate our main finding from Experiment One (facilitation of iMEP area with startle conditioning at 10 ms; see Results below) and to compare the number of observed iMEPs between the arm and hand. Again, we measured iMEPs in the dominant BB by delivering TMS over the ipsilateral M1 (non-dominant BB hotspot) at 100% maximum stimulator output with the target muscle pre-activated to ~20% of the participant’s MVC. TMS was delivered alone or 10 ms after the onset of a loud acoustic stimulus, with 16 trials for each condition (32 trials total) in a randomized order. Next, we measured cMEPs in the dominant BB (12 trials) by delivering TMS over the contralateral M1 (dominant BB hotspot) at the intensity required to elicit an ~0.5 mV motor evoked potential with the target muscle pre-activated to ~10% of the participant’s MVC. In a subgroup (n = 14), we measured iMEPs in the dominant FDI by delivering TMS over the ipsilateral M1 (non-dominant FDI hotspot) at 100% maximum stimulator output with the target muscle pre-activated to ~20% of the participant’s MVC. We delivered TMS alone or 10 ms after the onset of a loud acoustic stimulus, with 16 trials for each condition (32 trials total) in a randomized order. We also measured cMEPs in the dominant FDI (12 trials) by delivering TMS over the contralateral M1 (dominant FDI hotspot) at the intensity required to elicit an ~0.5 mV motor evoked potential with the target muscle pre-activated to ~10% of the participant’s MVC. For both cMEP and iMEP assessments, we provided visual feedback to the participant to assist maintenance of the target contraction. The inter-trial interval was ~10 s to reduce anticipation of stimuli and 30 s rest breaks were provided after every four trials.

#### 2.4.3. Experiment Three

In a third experiment, we compared the number of observed iMEPs between flexor and extensor muscles of the proximal and distal arm. We measured iMEPs in the dominant BB, TB, FCR and ECR by delivering TMS over the ipsilateral M1 (non-dominant hotspots) at 100% maximum stimulator output with the target muscle pre-activated to ~20% of the participant’s MVC. We delivered TMS alone or 10 ms after the onset of a loud acoustic stimulus, with 16 trials for each condition (32 trials total per muscle) in a randomized order. Next, we measured cMEPs in the dominant BB, TB, FCR and ECR (12 trials per muscle) by delivering TMS over the contralateral M1 (dominant hotspots) at the intensity required to elicit an ~0.5 mV motor evoked potential with the target muscle pre-activated to ~10% of the participant’s MVC. For both cMEP and iMEP assessments, we provided visual feedback to the participant to assist maintenance of the target contraction. The inter-trial interval was ~10 s to reduce anticipation of stimuli and 30 s rest breaks were provided after every four trials.

### 2.5. Data processing

We analyzed both iMEPs and cMEPs using previously described methods [15,18]. For each trial, we rectified the EMG and determined the mean background EMG 100 ms before stimulation. We determined the motor evoked potential onset latency as the time point when the poststimulus EMG exceeded the mean background EMG + 1 SD for at least 5 ms and the offset latency as the time point when the poststimulus EMG returned to this level [15,18]. For iMEPs, we measured onset latency within a predetermined window of 3–15 ms later than the onset latency of cMEPs [27]. We calculated the duration as the length between the onset and offset and determined the area of rectified EMG within this window. Group mean background EMG (absolute and MVC normalized), onset latency, and duration for both iMEPs and cMEPs in Experiment One, Two and Three can be found in Tables 1–3 respectively. We calculated motor evoked potential area using the area of rectified EMG in the duration window less an equivalent window of background EMG (mean background EMG x duration) [15]. We determined the mean iMEP and cMEP area for each participant. In Experiment One, all participants had at least eight iMEPs per condition. In Experiment Two, 7 out of 22 participants had less than eight iMEPs per condition and, therefore, were not included in the primary iMEP area analysis. In Experiment Two and Three, we determined the number of trials in which an iMEP was present for each muscle and condition. We used iMEP count rather than area as the primary dependent variable in Experiment Two and Three due to the low number of observed responses in all muscles other than BB. The same approach is used for other neurophysiological variables (e.g., F-waves) which are not typically elicited in every trial [26], therefore making analysis of size (i.e., amplitude or area) less reliable and inapropriate.

### 2.6. Statistical analysis

We performed all statistical analyses using SPSS software (Version 27; IBM, Armonk, NY). We assessed normality using Shapiro-Wilk’s test and homogeneity of variance using Levene’s test, which were not violated.

For Experiment One, we analyzed iMEP area and cMEP area (both n = 10) in the BB using separate one-way repeated measures ANOVAs with CONDITION (TMS alone, 1 ms, 5 ms, 10 ms, 15 ms) as the within-subjects factor. We made post hoc comparisons using paired t-tests adjusted using the Holm-Bonferroni correction. In the subgroup with cMEPs elicited in the BB using a lower TMS intensity, we compared cMEP area (n = 14) between conditions (TMS alone, 10 ms) using a paired *t*-test.

For Experiment Two, we compared iMEP count (n = 22) and area (n = 15) in the BB between conditions (TMS alone, 10 ms) using paired t-tests. In the subgroup, we compared iMEP count (n =14) in the arm (BB) and hand (FDI) using a two-way repeated measures ANOVA with EFFECTOR (arm, hand) and CONDITION (TMS alone, 10 ms) as the within-subjects factors.

For Experiment Three, we compared iMEP count (n = 15) in the flexor and extensor muscles of the proximal and distal upper limb using a three-way repeated measures ANOVA with ACTION (flexion, extension), LOCATION (proximal, distal) and CONDITION (TMS alone, 10 ms) as the within-subjects factors. We made post hoc comparisons using paired t-tests adjusted using the Holm-Bonferroni correction.

We set the significance level at p < 0.05. For ANOVAs and t-tests, we report partial eta squared ($\eta_{\text{p}}^{2}$) and Cohen’s d as a measure of effect size respectively. We present group data as mean ± SEM.

## 3. Results

### 3.1. Experiment One

**Fig. 2.**
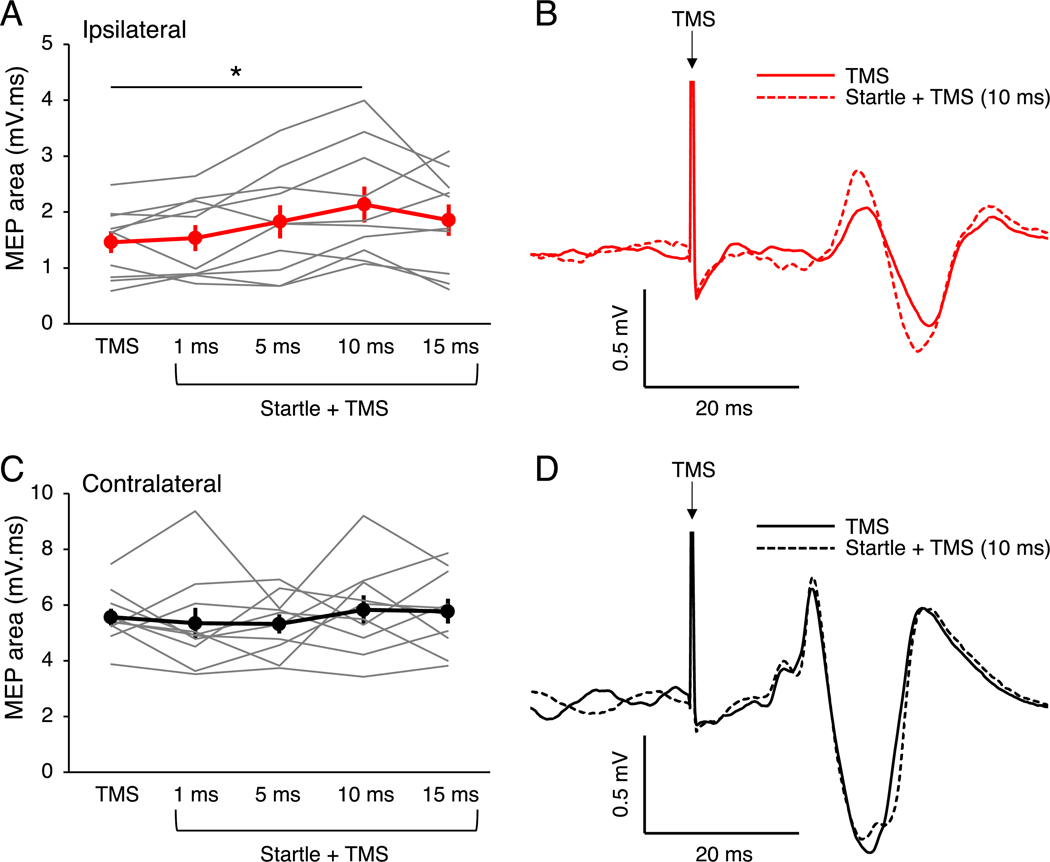
Ipsilateral and contralateral motor evoked potential (MEP) area in the arm. (A) Ipsilateral MEP area for the different conditions. Ipsilateral MEP area was larger with startle conditioning at 10 ms compared with transcranial magnetic stimulation (TMS) alone. (B) Example electromyography traces from an individual participant showing ipsilateral MEPs to TMS alone (solid) and with startle conditioning at 10 ms (dashed) in the biceps brachii muscle of the dominant arm. (C) Contralateral MEP area for the different conditions. Contralateral MEP area was not modulated by startle conditioning at any interval. (D) Example electromyography traces from an individual participant showing contralateral MEPs to TMS alone (solid) and with startle conditioning at 10 ms (dashed) in the biceps brachii muscle of the dominant arm. For panels A and C, the graphs show the group mean ± SEM and individual data. *p < 0.05.

In our first experiment, we tested whether startle conditioning modulates the size of iMEPs and cMEPs. For iMEP area, there was a main effect of CONDITION (F_4,36_ = 5.57, p = 0.001, $\eta_{\text{p}}^{2}$ = 0.38), with iMEP area being greater with startle-conditioned TMS at the 10 ms interval compared to TMS alone (t_9_ = 3.65, p = 0.021, d = 0.81; Fig. 2A and B). There were no significant differences in iMEP area between startle-conditioned TMS at the 1, 5 or 15 ms intervals and TMS alone (all p > 0.103). For cMEP area, there was no main effect of CONDITION (F_4,36_ = 0.63, p = 0.643, $\eta_{\text{p}}^{2}$ = 0.07; Fig. 2C and D). The lack of modulation of cMEPs with startle conditioning is unlikely to be due to a ceiling effect as there was no difference in cMEP area between startle-conditioned TMS at 10 ms (2.13 ± 0.21 mV ms) and TMS alone (2.07 ± 0.24 mV ms, t_13_ = 0.30, p = 0.766) using the lower TMS intensity.

### 3.2. Experiment Two

**Fig. 3.**
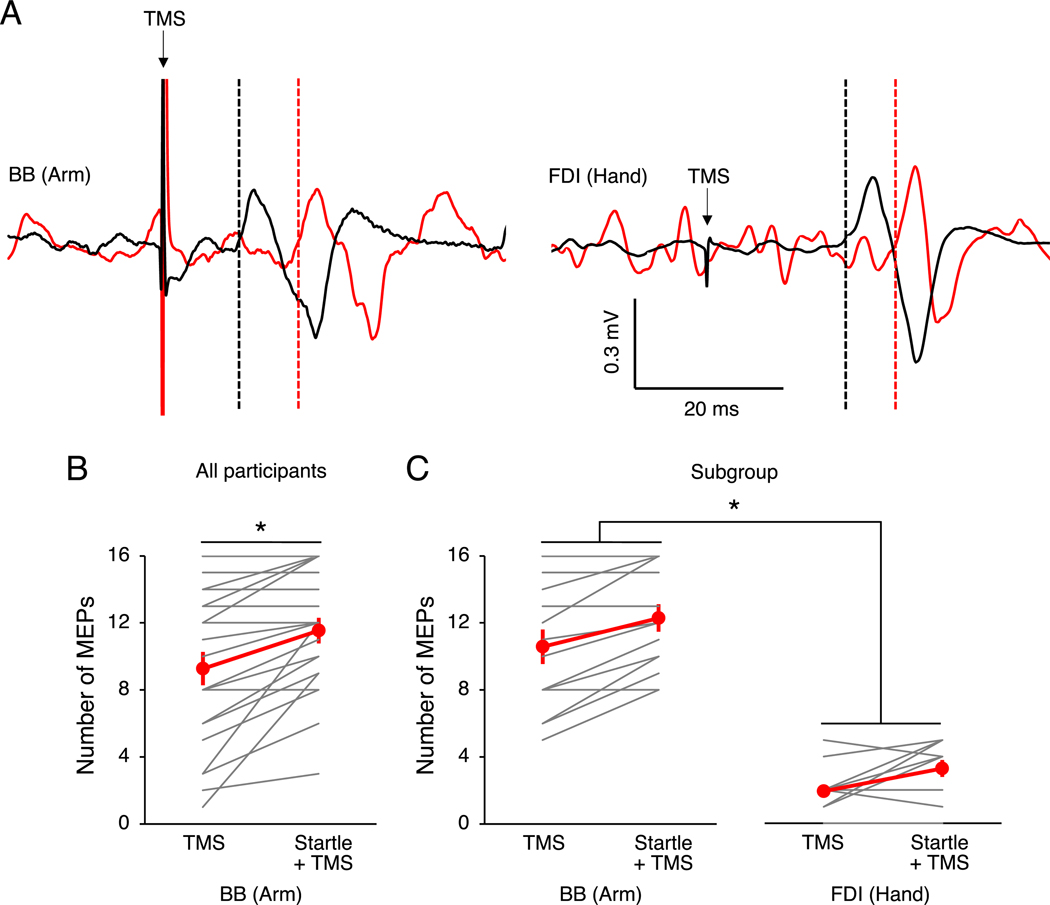
Ipsilateral motor evoked potential (MEP) presence in the arm and hand. (A) Example electromyography traces from an individual participant showing contralateral (black) and ipsilateral (red) motor evoked potentials to transcranial magnetic stimulation (TMS) alone in the biceps brachii (BB) and first dorsal interosseous (FDI) muscles of the dominant arm. Dashed lines indicate onset latencies. (B) The number of observed ipsilateral MEPs with TMS alone and with startle conditioning at 10 ms in the BB muscle of the arm in all participants. Ipsilateral MEP count was higher with startle conditioning compared with TMS. (C) The number of observed ipsilateral MEPs with TMS alone and with startle conditioning at 10 ms in the BB muscle of the arm and FDI muscle of the hand in a subgroup of participants. For both muscles, ipsilateral MEP count was higher with startle conditioning compared with TMS alone, but more ipsilateral MEPs were observed in the BB compared with the FDI. For panels B and C, the graphs show the group mean ± SEM and individual data. *p < 0.05. (For interpretation of the references to colour in this figure legend, the reader is referred to the Web version of this article.)

In our second experiment, we first sought to replicate our main finding from Experiment One (facilitation of iMEPs with startle conditioning at 10 ms). Consistent with Experiment One, iMEP area was greater with startle-conditioned TMS (1.99 ± 0.26 mV ms) compared to TMS alone (1.63 ± 0.20 mV ms; t_14_ = 3.29, p = 0.005, d = 0.36). Importantly, the facilitatory effect of startle conditioning also extended to iMEP count, as more iMEPs were present with startle-conditioned TMS compared to TMS alone (t_21_ = 4.35, p < 0.001, d = 0.55; Fig. 3B).

In the subgroup, we compared iMEP count between the arm and the hand. For iMEP count, there was a main effect of EFFECTOR (F_1,13_ = 109.96, p < 0.001, $\eta_{\text{p}}^{2}$ = 0.89) and CONDITION (F1,13 = 30.16, p < 0.001, $\eta_{\text{p}}^{2}$ =0.70), but no interaction (F1,13 =0.29, p =0.597, $\eta_{\text{p}}^{2}$ =0.02). We found that more iMEPs were present in the arm compared with the hand (Fig. 3C).

### 3.3. Experiment Three

**Fig. 4.**
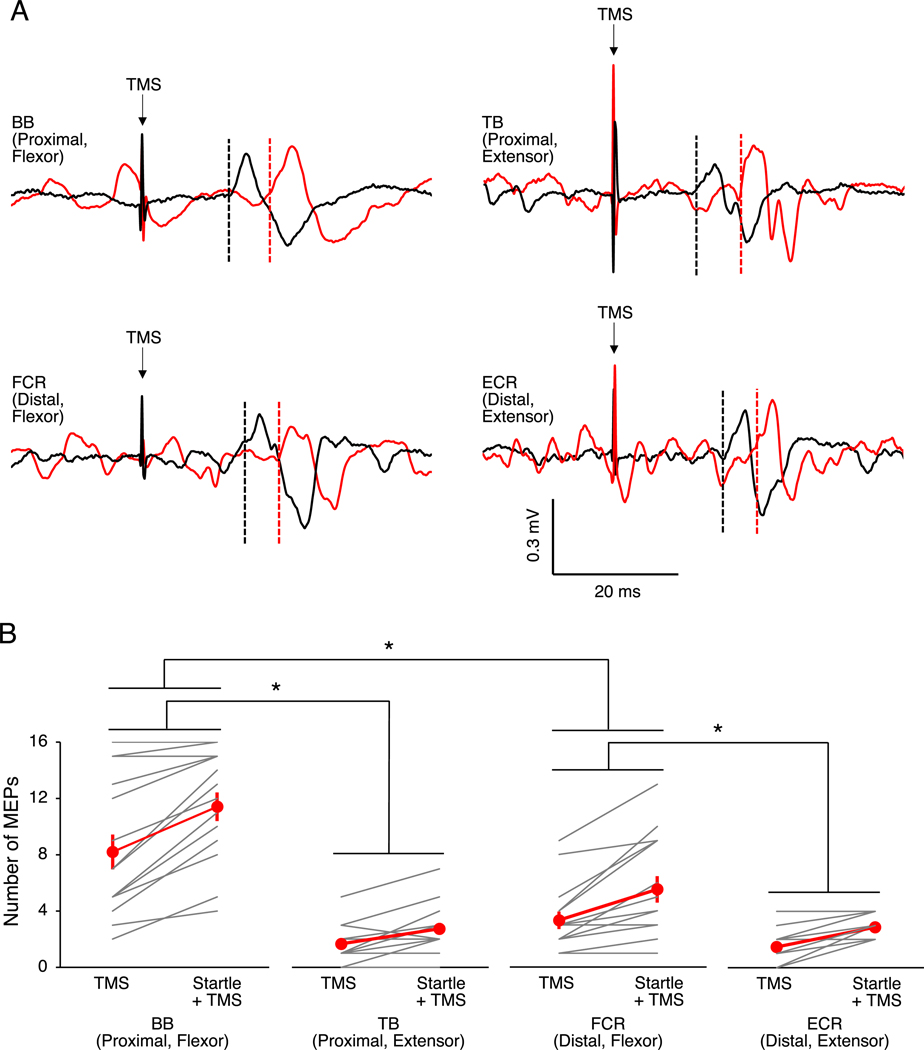
Ipsilateral motor evoked potential (MEP) presence in flexors and extensors of the proximal and distal arm. (A) Example electromyography traces from an individual participant showing contralateral (black) and ipsilateral (red) motor evoked potentials to transcranial magnetic stimulation (TMS) alone in the biceps brachii (BB), triceps brachii (TB), flexor carpi radialis (FCR) and extensor carpi radialis (ECR) muscles of the dominant arm. Dashed lines indicate onset latencies. (B) The number of observed ipsilateral MEPs with TMS alone and with startle conditioning at 10 ms in the BB, TB, FCR and ECR muscles of the arm. For all muscles, ipsilateral MEP count was higher with startle conditioning compared with TMS. Ipsilateral MEP count was higher in the proximal flexor (BB) compared with the proximal extensor (TB) and distal flexor (FCR) and was higher in the distal flexor (FCR) compared with the distal extensor (ECR). For panel B, the graphs show the group mean ± SEM and individual data. *p < 0.05. (For interpretation of the references to colour in this figure legend, the reader is referred to the Web version of this article.)

In our third experiment, we compared iMEP count between flexor and extensor muscles of the proximal and distal arm. For iMEP count, there was a main effect of ACTION (F_1,14_ = 45.21, p < 0.001, $\eta_{\text{p}}^{2}$ = 0.76), LOCATION (F_1,14_ = 75.51, p < 0.001, $\eta_{\text{p}}^{2}$ = 0.84) and CONDITION (F_1,14_ = 97.18, p < 0.001, $\eta_{\text{p}}^{2}$ = 0.87). There was also a ACTION x LOCATION (F_1,14_ = 24.80, p < 0.001, $\eta_{\text{p}}^{2}$ = 0.64) and ACTION x CONDITION (F_1,14_ = 11.23, p = 0.005, $\eta_{\text{p}}^{2}$ = 0.45) interaction, but no other interactions (both F_1,14_ < 1.77, p *>* 0.205, $\eta_{\text{p}}^{2}$ < 0.11). Post-hoc comparisons indicated that iMEP count was higher in BB compared with TB (t_14_ = 7.04, p < 0.001, d = 2.41; Fig. 4B) and FCR (t_14_ = 6.78, p < 0.001, d = 1.47). Also, iMEP count was higher in FCR compared with ECR (t_14_ = 3.25, p = 0.012, d = 1.05). Interestingly, there was no difference in iMEP count between TB and ECR (t_14_ = 0.09, p = 0.930, d = 0.03). Together, these findings indicate that more iMEPs were present in proximal and flexor muscles compared with distal and extensor muscles of the arm.

## 4. Discussion

Here, we show that pairing a startling acoustic stimulus with TMS over ipsilateral M1 increases the size and presence of iMEPs. Using this novel startle-conditioned TMS technique, we found that iMEPs are more prominent in proximal compared with distal, as well as flexor compared with extensor muscles of the upper limb. Given the known characteristics of the reticulospinal tract, these findings indicate that this pathway mediates iMEPs. Importantly, pairing startle stimuli with TMS over ipsilateral M1 can become a useful method to track the modulation of cortico-reticulospinal projections following interventions and in neurological conditions where the reticulospinal tract may be involved in motor recovery.

To date, non-invasive assessment of the reticulospinal tract in awake humans has proven difficult. Previous research has described the presence of iMEPs in the arm to TMS over ipsilateral M1, and suggested that the primary pathway mediating iMEPs was either the small uncrossed portion (~10%) of the corticospinal tract [17], or corticofugal fibers connecting with the ipsilateral reticular formation in the brainstem and then to motoneurons in the spinal cord (i.e., the reticulospinal tract) [15, 18]. Elucidating whether the reticulospinal tract mediates iMEPs is important, because it could become a useful method to non-invasively assess reticulospinal excitability in humans.

Combining startle conditioning with TMS increased the size and number of observed iMEPs. Delivering a loud acoustic stimulus can evoke a startle response in humans [13]. Evidence in animals indicates the startle response is mediated by the pontomedullary reticular formation in the brainstem [14], with responses in this region to startling acoustic stimuli recorded at very short latencies (~3 ms) [25]. In the present study, robust facilitation of iMEPs was observed in humans when delivering a startling acoustic stimulus 10 ms prior to TMS over ipsilateral M1. This interval coincides with the latency of the auditory brainstem reponse in humans (5–10 ms) [24], and may reflect the optimal timing in which the startling acoustic stimulus raises the excitability of the reticular formation before this subcortical structure is activated by descending projections from ipsilateral M1.

We observed no modulation of cMEPs which primarily reflect direct corticospinal excitability [16]. Previous studies have demonstrated that delivering a startle stimulus 30–60 ms prior to TMS over M1 leads to suppression of cMEPs [19–22]. Consistent with our data, no modulation of cMEPs was observed when the startle stimulus was delivered at shorter intervals (<20 ms) [19]. Suppresion of cMEPs is thought to reflect inhibition of motor cortical areas by ascending projections from the reticular formation via the thalamus [19]. Interestingly, motor evoked potentials to direct activation of the corticospinal tract at the cervicomedullary junction with electrical stimulation are facilitated at longer (80 ms), but not shorter (<20 ms), intervals. This late facilitation is thought to reflect raised spinal excitability [19,21]. Therefore, the iMEP facilitation observed at 10 ms in the present study is unlikely to be mediated by excitability changes at the cortical or spinal level. Also, it is unlikley that the modulation of iMEPs at 10 ms reflect any behavioral overt startle response. This is because the onset latency for the iMEPs (~20 ms) was a lot shorter than the onset latency of muscle activity in the upper limb in response to the acoustic startle stimuli alone (~80 ms) previously reported [13].

We found that iMEPs were more prominent in proximal than distal muscles of the upper limb. Specifically, more iMEPs were elicited in the proximal arm (BB) compared with both the distal forearm (FCR) and hand (FDI). Our findings are consistent with studies in non-human primates which indicated that the reticulospinal tract primarily innervates axial and proximal effectors [9,10]. Though, projections to motoneurons of distal upper limb effectors (forearm and hand) have also been observed [6–8]. The greater prominence of iMEPs (i.e., the reticulospinal tract) towards proximal upper limb effectors observed here could explain in part the recovery of strength in the arm, but not the hand, observed after severe corticospinal tract damage poststroke in humans [28].

We also found that iMEPs were more prominent in flexor than extensor muscles of the upper limb. Greater prominence of iMEPs in flexors was evident at both the proximal arm (BB vs. TB) and distal forearm (FCR vs. ECR). Again, this pattern is consistent with findings in non-human primates indicating that the reticulospinal tract innervation of flexor muscles is greater than extensors [8]. Of note, further imbalancing of reticulospinal inputs towards flexor muscles is evident after a lesion to the corticospinal tract in monkeys [11]. Thus, the features of iMEPs found here might partially explain the development of intrusive abnormal flexor synergies (i.e., loss of independent joint control) following corticospinal tract damage after stroke in humans [29–31].

The present study has some limitations. The size and presence of iMEPs in effectors across the upper limb were only assessed at a single voluntary contraction level (20% of MVC). In non-human primates, the firing rate of cells in the reticular formation increases linearly with contraction force [32]. Whether greater prominence of iMEPs in proximal and flexor muscles observed in our study is also evident at higher contraction levels remains to be determined. It is important to note, however, that a low voluntary contraction level was selected in the present study as these conditions are relevant for measuring iMEPs in neurologically impaired individuals with profound upper limb weakness, such as after stroke [28]. Also, based on the findings from Experiment One, a single, fixed interval between the onset of the loud acoustic stimulus and TMS (10 ms) was used in Experiment Two and Three. Assessment of iMEPs using startle-conditioned TMS might be further optimized by adjusting the interstimulus interval to account for inter-individual variability in conduction velocity and propogation distance of the startling auditory tone. Although time consuming, these methodological factors may be particularly relevant when using startle-conditioned TMS to measure iMEPs in aging and clinical cohorts.

In summary, we show that pairing a startling acoustic stimulus with TMS over ipsilateral M1 increases the size and presence of iMEPs. Using this novel startle-conditioned TMS technique, we demonstrate that iMEPs are more prominent in proximal compared with distal, as well as flexor compared with extensor muscles of the upper limb. Together, these properties strongly suggest that the reticulospinal tract is the primary pathway mediating iMEPs. Our findings have important implications for tracking the modulation of cortico-reticulospinal projections following interventions, such as strength training [33], and in neurological conditions where the reticulospinal tract may be involved, such as in motor recovery following stroke [28].

## Supplementary Material

1

## Figures and Tables

**Table 1:** Additional TMS variables for Experiment One.

	TMS alone	1 ms	5 ms	10 ms	15 ms
*iMEPs* rmsEMG (mV)	0.13 (0.01)	0.14 (0.01)	0.13 (0.01)	0.14 (0.01)	0.14 (0.01)
Activation (% MVC)	20.3 (0.9)	22.1 (0.9)	19.9 (1.1)	21.0 (0.6)	21.3 (0.9)
Latency (ms)	20.0 (0.8)	20.2 (0.4)	20.3 (0.5)	19.5 (0.5)	19.8 (0.7)
Duration (ms)	12.2 (0.4)	12.2 (0.7)	12.5 (0.5)	13.0 (0.6)	12.0 (0.7)
*cMEPs* rmsEMG (mV)	0.12 (0.01)	0.11 (0.01)	0.11 (0.01)	0.11 (0.01)	0.12 (0.01)
Activation (% MVC)	18.3 (1.3)	18.1 (1.4)	17.5 (1.3)	17.8 (1.6)	18.2 (1.4)
Latency (ms)	12.0 (0.3)	12.2 (0.3)	12.1 (0.2)	12.1 (0.2)	12.2 (0.3)
Duration (ms)	12.7 (0.6)	12.5 (0.7)	12.5 (0.7)	12.9 (0.7)	12.6 (0.7)
*cMEPs (0.*5 mV) rmsEMG (mV)	0.07 (0.01)	–	–	0.07 (0.01)	–
Activation (% MVC)	9.8 (1.2)	–	–	9.3 (1.1)	–
Latency (ms)	12.9 (0.5)	–	–	13.0 (0.6)	–
Duration (ms)	12.1 (0.8)	–	–	11.9 (0.8)	–

**Note:** Data are shown as group mean (SEM). TMS – transcranial magnetic stimulation, iMEPs – ipsilateral motor evoked potentials, rmsEMG – root mean square electromyography, MVC – maximum voluntary contraction, cMEPs – contralateral motor evoked potentials.

**Table 2:** Additional TMS variables for Experiment Two.

	TMS alone	10 ms
*BB iMEPs* rmsEMG (mV)	0.10 (0.01)	0.10 (0.01)
Activation (% MVC)	21.8 (0.5)	21.5 (0.4)
Latency (ms)	19.8 (0.4)	19.0 (0.3)
Duration (ms)	12.1 (0.4)	13.5 (0.3)
*BB cMEPs* rmsEMG (mV)	0.04 (0.01)	–
Latency (ms)	12.1 (0.2)	–
Duration (ms)	14.0 (0.4)	–
*FDI iMEPs* rmsEMG (mV)	0.12 (0.01)	0.12 (0.01)
Activation (% MVC)	22.0 (0.5)	23.0 (1.0)
Latency (ms)	27.1 (0.7)	29.0 (0.8)
Duration (ms)	8.5 (0.5)	8.4 (0.2)
*FDI cMEPs* rmsEMG (mV)	0.03 (0.01)	–
Latency (ms)	21.3 (0.4)	–
Duration (ms)	9.5 (0.5)	–

**Note:** Data are shown as group mean (SEM). TMS – transcranial magnetic stimulation, BB – biceps brachii, iMEPs – ipsilateral motor evoked potentials, rmsEMG – root mean square electromyography, MVC – maximum voluntary contraction, cMEPs – contralateral motor evoked potentials, FDI – first dorsal interosseous.

**Table 3:** Additional TMS variables for Experiment Three.

	BB	TB	FCR	ECR
*iMEPs (TMS alone)* rmsEMG (mV)	0.10 (0.01)	0.06 (0.01)	0.08 (0.01)	0.07 (0.01)
Activation (% MVC)	20.5 (1.0)	21.8 (1.1)	22.0 (1.6)	20.6 (1.3)
Latency (ms)	21.3 (0.5)	19.3 (0.5)	22.5 (0.7)	23.4 (0.5)
Duration (ms)	15.2 (0.6)	11.3 (0.9)	10.1 (0.4)	9.5 (0.7)
*iMEPs* (10 ms) rmsEMG (mV)	0.10 (0.01)	0.06 (0.01)	0.08 (0.01)	0.07 (0.01)
Activation (% MVC)	20.6 (1.2)	22.5 (1.0)	21.4 (1.4)	20.8 (1.4)
Latency (ms)	20.5 (0.6)	20.4 (0.9)	21.8 (0.5)	23.1 (0.7)
Duration (ms)	16.6 (0.5)	11.7 (0.8)	10.5 (0.4)	9.6 (0.6)
*cMEPs* rmsEMG (mV)	0.02 (0.01)	0.02 (0.01)	0.02 (0.01)	0.02 (0.01)
Latency (ms)	12.6 (0.3)	12.3 (0.2)	15.6 (0.2)	15.9 (0.2)
Duration (ms)	15.9 (0.5)	13.7 (1.1)	12.5 (0.7)	12.8 (0.4)

**Note:** Data are shown as group mean (SEM). TMS – transcranial magnetic stimulation, BB – biceps brachii, TB – triceps brachii, FCR – flexor carpi radialis, ECR – extensor carpi radialis, iMEPs – ipsilateral motor evoked potentials, rmsEMG – root mean square electromyography, MVC – maximum voluntary contraction, cMEPs – contralateral motor evoked potentials.
