# Supplementary material for: Mapping subcortical motor pathways in humans with startle-conditioned TMS
Source: Brain Stimul. Author manuscript; Available in PMC 2025 Jan 12. (PMC11724745; doi:10.1016/j.brs.2023.08.010)
Supplement: 1 [file NIHMS1941404-supplement-1.docx]

**Supplementary material**

**Reliability of iMEPs**

In a subgroup of participants (n = 8) from Experiment Two, we remeasured ipsilateral motor evoked potentials (iMEPs) in the dominant *biceps brachii* (BB) after ~30 min, during which time participants were seated at rest. We determined the intra-day measurement error for iMEP count and area, elicited with and without startle conditioning, by quantifying the standard error of the measurement (SEMeas). For iMEP area, we only included participants with at least eight iMEPs (n = 4 participants for TMS alone, n = 6 participants for 10 ms). We calculated the SEMeas by taking the square root of the within-subject variance partitioned by a one-way ANOVA with SUBJECT as the factor [[1](#_ENREF_1), [2](#_ENREF_2)]. We also calculated the relative SEMeas for each by normalizing the absolute SEMeas to the group mean, allowing us the compare the measurement error of iMEP count and area with other TMS variables. From the SEMeas, we calculated the intra-day smallest detectable change (SDC) for an individual participant using the following formula [[1](#_ENREF_1), [2](#_ENREF_2)]:

$$SDC=SEMeas \times\sqrt{m} \times1.96$$

where ‘m’ is the number of measurements made per participant and 1.96 represents the 95% confidence interval. The SDC provides a boundary to which an outcome should increase or decrease by in order to conclude, with high confidence, that the observed change exceeds what is expected due to measurement error.

The absolute and relative SEMeas and the SDC are shown in Table S1. The within-session measurement error for iMEP count and area in BB was low, ranging from 10-16%. Measurement error, informs the degree to which repeated measurements vary, with lower values indicating that the measure will more likely be sensitive to change [[3](#_ENREF_3)]. The measurement error of iMEP count and area is similar to other commonly used TMS measures (e.g., motor threshold) [[3](#_ENREF_3)]. From the measurement error, we determined SDC estimates for within-session measurements. The SDC provides a threshold which can be used to determine if modulation is greater than what might be expected due to “measurement noise”. Low measurment error of iMEP count and area supports their utility for tracking within-session changes in cortico-reticulospinal excitability. Future experimenters could use the SDC estimates generated here to determine the degree of modulation required to be considered a true change in cortico-reticulospinal excitability.

**Table S1.** Reliability of iMEPs.

|  | Absolute SEMeas | Relative SEMeas | SDC |
| --- | --- | --- | --- |
| *TMS alone* |  |  |  |
| Count | 1 | 16% | 3 |
| Area | 0.21 mV.ms | 10% | 0.57 mV.ms |
| *10 ms* |  |  |  |
| Count | 1 | 10% | 3 |
| Area | 0.35 mV.ms | 14% | 0.98 mV.ms |

**Note:** iMEPs – ipsilateral motor evoked potentials, SEMeas – standard error of the measurement, SDC – smallest detectable change, TMS – transcranial magnetic stimulation.

**Comparison of background EMG**

We compared absolute and maximum voluntary contraction (MVC) normalized background electromyography (EMG) between conditions to ensure that differences in activation of the target muscle(s) did not account for the greater size and presence of iMEPs in the startle TMS condition compared with TMS alone. For Experiment One, we analyzed iMEP trial background EMG (n = 10) and cMEP trial background EMG (n = 10) in the BB using separate one-way repeated measures ANOVAs with CONDITION (TMS alone, 1 ms, 5 ms, 10 ms, 15 ms) as the within-subjects factor. In the subgroup with cMEPs elicited using a lower TMS intensity, we compared cMEP trial background EMG (n = 14) in the BB between conditions (TMS alone, 10 ms) using a paired t-test. There was no difference in absolute or MVC normalized background EMG between conditions for iMEP trials or cMEP trials (all p > 0.116). For Experiment Two, we compared iMEP trial background EMG (n = 22) in the BB between conditions (TMS alone, 10 ms) using a paired t-test. In the subgroup, we compared iMEP trial background EMG (n = 14) using a two-way repeated measures ANOVA with EFFECTOR (arm, hand) and CONDITION (TMS alone, 10 ms) as the within-subjects factors. There was no difference in absolute or MVC normalized background EMG between conditions (all p > 0.120). For Experiment Three, we compared iMEP trial background EMG (n = 15) using a three-way repeated measures ANOVA with ACTION (flexion, extension), LOCATION (proximal, distal) and CONDITION (TMS alone, 10 ms) as the within-subjects factors. There was no difference in absolute or MVC normalized background EMG between conditions (all p > 0.168).

**References**

[1] Samusyte G, Bostock H, Rothwell J, Koltzenburg M. Short-interval intracortical inhibition: Comparison between conventional and threshold-tracking techniques. Brain Stimul 2018;11(4):806-17.

[2] Mooney RA, Casamento-Moran A, Celnik PA. The reliability of cerebellar brain inhibition. Clin Neurophysiol 2021;132(10):2365-70.

[3] Beaulieu LD, Flamand VH, Masse-Alarie H, Schneider C. Reliability and minimal detectable change of transcranial magnetic stimulation outcomes in healthy adults: A systematic review. Brain Stimul 2017;10(2):196-213.
